# Regeneration of the Nerves in the Aerial Cavity with an Artificial Nerve Conduit -Reconstruction of Chorda Tympani Nerve Gaps-

**DOI:** 10.1371/journal.pone.0092258

**Published:** 2014-04-01

**Authors:** Toshiaki Yamanaka, Hiroshi Hosoi, Takayuki Murai, Takehiko Kobayashi, Yuji Inada, Tatsuo Nakamura

**Affiliations:** 1 Department of Otolaryngology-Head and Neck Surgery, Nara Medical University School of Medicine, Nara, Japan; 2 Department of Orthopedic surgery, Inada Hospital, Nara, Japan; 3 Department of Bioartificial Organs. Institute for Frontier Medical Science, Kyoto University, Kyoto, Japan; Duke University Medical Center, United States of America

## Abstract

**Objectives/Hypothesis:**

Due to its anatomical features, the chorda tympani nerve (CTN) is sometimes sacrificed during middle ear surgery, resulting in taste dysfunction. We examined the effect of placing an artificial nerve conduit, a polyglycolic acid (PGA)-collagen tube, across the gap in the section of the resected chorda tympani nerve (CTN) running through the tympanic cavity.

**Methods:**

The CTN was reconstructed with a PGA-collagen tube in three patients with taste disturbance who underwent CTN resection. To evaluate the effect of the reconstruction procedure on the patients' gustatory function, we measured the patients' electrogustometry (EGM) thresholds. The patients were followed-up for at least two years.

**Results:**

Gustatory function was completely restored in all of the patients after the reconstruction. The patients' EGM thresholds exhibited early improvements within one to two weeks and had returned to their normal ranges within three months. They subsequently remained stable throughout the two-year follow-up period. In a patient who underwent a second surgical procedure, it was found that the PGA-collagen tube used in the first surgical procedure had been absorbed and replaced by new CTN fibers with blood vessels on their surfaces.

**Conclusion:**

These results suggest that reconstruction of the CTN with an artificial nerve conduit, a PGA-collagen tube, allows functional and morphological regeneration of the nerve and facilitates the recovery of taste function. PGA-collagen tubes might be useful for repairing CTNs that are resected during middle ear surgery. Further research is required to confirm these preliminary results although this is the first report to describe the successful regeneration of a nerve running through an aerial space.

## Introduction

The chorda tympani nerve (CTN) contains primary afferent nerve fibers that transmit taste information signals from the anterior two thirds of the tongue to the solitary nucleus in the medulla oblongata. The CTN leaves the facial nerve and runs through an aerial space of the middle ear [1]. Thus, it is frequently severed during middle ear surgery for some diseases involving otitis media and cholesteatoma, etc. [2]. Resection of the CTN causes defects in gustatory function on the affected side, resulting in taste disturbance in most cases [3], [4].

Restoring severed nerves with achieving sufficient functional recovery continues to be a clinical challenge. Attempts have been to use various artificial nerve conduits as alternatives to autografts for regenerating severed peripheral nerves [5], [6], [7]. A bioabsorbable polyglycolic acid (PGA) tube filled with collagen sponge (PGA-collagen tube) has recently been developed since PGA tube without luminal matrix was initially used for the nerve repair [8], and was confirmed to aid the regeneration of peripheral nerve defects in our experimental and clinical trials [9]. However, there are no reports about the application of the device to CTN reconstruction. When resected, it is difficult to restore the CTN because it runs through an aerial space without any floor beneath it. PGA tube is considered to act as a scaffold for nerve regeneration [9]. Therefore, we attempted to repair nerve gaps using a PGA-collagen tube in a patient who underwent CTN section during middle ear surgery. In addition, we also examined whether the procedure cured the patient's gustatory dysfunction.

## Materials and Methods

This study was approved by the Institutional Review Board at Nara Medical University Hospital. Participants provided their written informed consent to participate in this study. There are no financial interests or potential conflict of interests in this study.

Three patients who preoperatively presented with dysgeusia (feeling decreased sweetness) were included in this study. Patient 1 was a 52-year-old male who presented with a three-month history of taste disturbance due to a cholesteatoma involving the CTN. Patient 2 was a 33-year-old male who presented with taste disturbance due to a temporal bone fracture that had lasted for three months. Patient 3 was an 18-year-old female with a cholesteatoma who had a five-month history of unilateral gustatory dysfunction. Reconstruction of the CTN with an artificial nerve conduit, a polyglycolic acid (PGA)-collagen tube, was performed for each patient who underwent the resection of CTN that was strongly affected by the lesion (cholesteatoma sac or ossicles dislocation). To evaluate the effect of the reconstruction on the patient's gustatory function, we measured the electrogustometry (EGM) threshold. The patients were followed-up over at least two years, although patient 1 had never come to our clinic in a period ranging from 4–17 months after the surgery.

### Design of the PGA collagen tube

**Figure 1:**
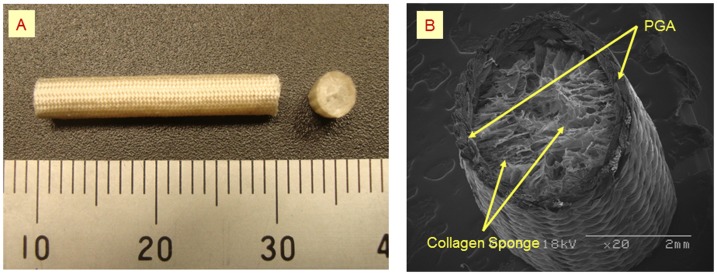
Polyglycolic acid (PGA) tube filled with collagen sponge (PGA-collagen tube) as a nerve guide. (A) PGA-collagen tube and its transverse section. (B) Scanning electron microscopic view of the transverse section of PGA-collagen tube.

The artificial nerve conduit was composed of a biodegradable tube filled with biodegradable filaments. The tube framework was made of a cylindrically woven PGA mesh, the outer and inner surfaces of which were coated with amorphous collagen layers. Microscopic appearance and ultrastructure of the conduit as observed through scanning electron microscopy (SEM) are shown in Figure 1. The lumen of the tube was filled with a 3D spongiform collagen matrix. Spongiform collagen, which has a greater surface area than fibrous collagen, seems to provide a favorable microenvironment for nerve progression (axonal sprouting, cellular proliferation, and tissue healing) [10], [11]. Although collagen has a tendency to dissolve in the body, the PGA coating is considered to allow the tube to retain its shape for a sufficiently long period for complete nerve regeneration to occur [10], [12]. The PGA-collagen tube (30 mm in length, 0.7 mm in diameter) was sterilized prior to surgery using ethylene oxide gas and trimmed to appropriate length during surgery. The detailed method used to prepare the nerve conduit was described in our previous article [10], [11], [12], [13], [14].

### Surgical procedure

**Figure 2:**
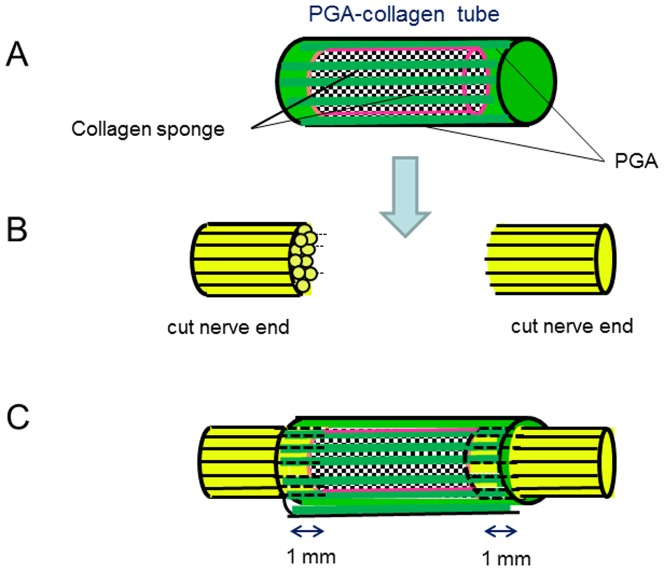
Schema of the reconstruction of chorda tympani nerve with PGA-collagen tube. (A) PGA-collagen tube was trimmed to appropriate length (2 mm longer than the gap) to fit the nerve gap. (B) Both the proximal and distal stumps of the severed nerve were inserted into the lumen of the tube filled with a collagen sponge matrix to a depth of 1 mm. (C) Interposition of PGA-collagen tube between nerve cut ends.

The surgery was performed under surgical microscope with retroauricular incision. The nerve gaps produced by the resection of the CTN measured seven, five, and seven mm in length in patients 1, 2, and 3, respectively. PGA-collagen tube was trimmed to appropriate length (two mm longer than the gap) to fit the nerve gap (Figure 2A). The resultant nerve gap was reconstructed with a PGA-collagen tube. Both the proximal and distal stumps of the severed nerve were inserted into the lumen of the tube filled with a 3D spongiform collagen matrix to a depth of one mm (Figure 2B), and the tube was secured to the proximal and distal nerve ends with epineural sutures without any floor for a scaffold in an aerial space (Figure 2C).

### Outcome measures

Gustatory function was assessed with electrogustometry (EGM) using an electrogustometer (Rion TR-06, Rion Co, Tokyo, Japan), which is a more reliable and objective method of quantitatively evaluating taste function [15], [16]. During the EGM, a five-mm probe was applied to the lateral edge of the tongue at two cm from the tip to stimulate the CTN territory. The stimulation range of the EGM was −8 to 34 dB (normal range: ≤8 dB). Scale out, which was not detected at any EGM stimulation intensity, was taken as 36 dB.

## Results

**Figure 3:**
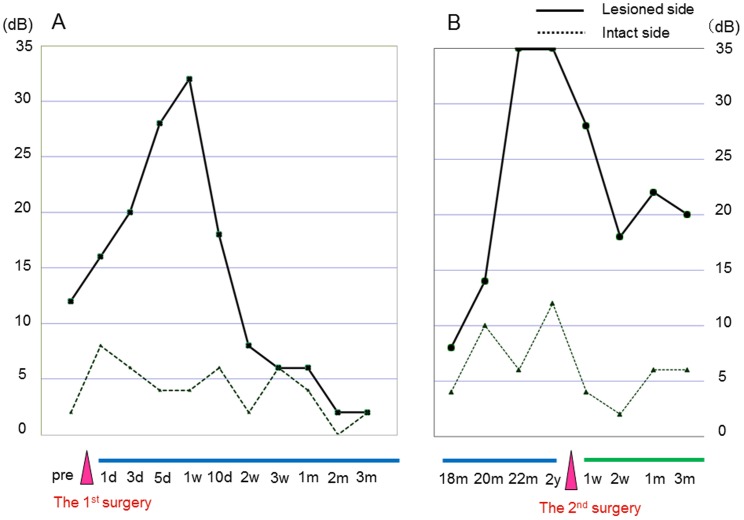
The EGM threshold after reconstruction of the CTN with a PGA-collagen tube in patient 1. Abbreviations: pre, preoperative period; d, day; w, week; m, month; y, year.

**Figure 4:**
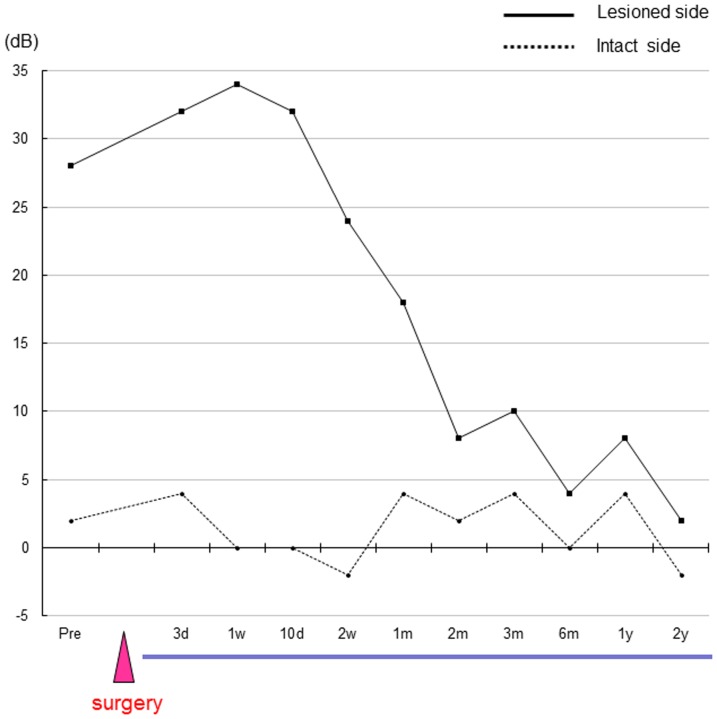
The EGM threshold after reconstruction of the CTN with a PGA-collagen tube in patient 2. Abbreviations: pre, preoperative period; d, day; w, week; m, month; y, year.

**Figure 5:**
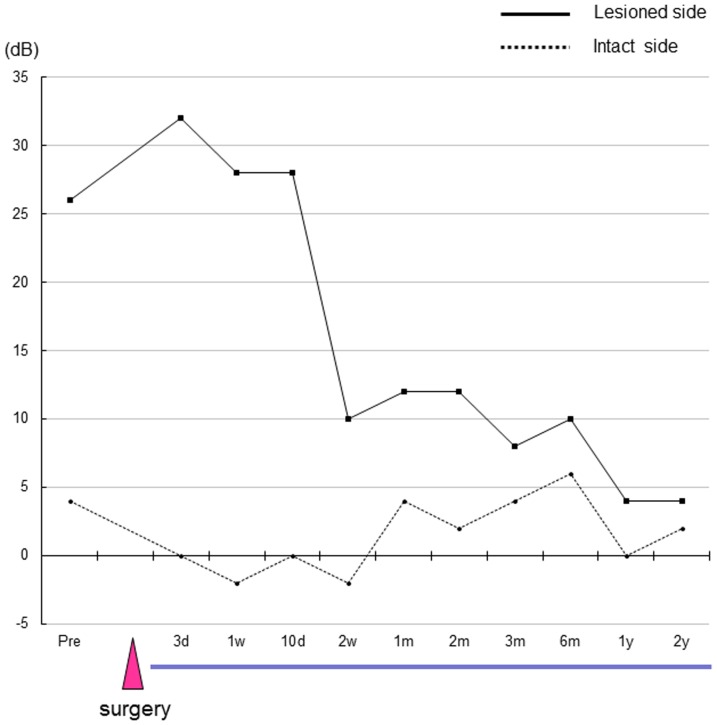
The EGM threshold after reconstruction of the CTN with a PGA-collagen tube in patient 3. Abbreviations: pre, preoperative period; d, day; w, week; m, month; y, year.

The nerve gaps produced by the resection of the CTN measured approximate seven mm in length in all three patients. The patients' EGM thresholds before and after CTN reconstruction with the PGA-collagen tube are shown in Figure 3, 4 and 5. The EGM thresholds of patients 1, 2, and 3 were 12, 28 and 26 dB, respectively, before surgery and had increased to 32, 34, and 32 dB, respectively, within one week after CTN resection and reconstruction. Thereafter, these measurements started to improve in a period ranging from one to two weeks in all patients. The patients' EGM threshold had reached the normal range within two weeks in patient 1 (Figure 3A), 2 months in patient 2 (Figure 4), and 3 months in patient 3 (Figure 5). These improvements remained unchanged at 18 postoperative months in patient 1 and at 2 postoperative years in patients 2 and 3 although patient 1, whose case is described as an index case, developed gustatory dysfunction due to cholesteatoma recurrence at 22 postoperative months (Figure 3B). In contrast, the EGM threshold on the intact side showed little change throughout the patients' postoperative course.

On the other hands, the patients' sweet taste disturbance as a primary subjective symptom had reached the normal feeling by 2 postoperative weeks in patient 1, three months in patient 2, and one month in patient 3.

No adverse events including middle or inner ear dysfunction were observed in any of the patients.

### An index Case

A 52-year-old male presented with a three-month history of taste disturbance (feeling decreased sweetness on the right side of his tongue) and a two-month history of hearing loss in his right ear. A physical examination detected a white cholesteatoma mass and the formation of inflammatory granulation tissue in the pars flaccida over the manubrium of the malleus. A CT scan obtained with the bone window setting demonstrated the presence of a soft-tissue mass in the epitympanum and tympanic antrum together with ossicular erosion.

**Figure 6:**
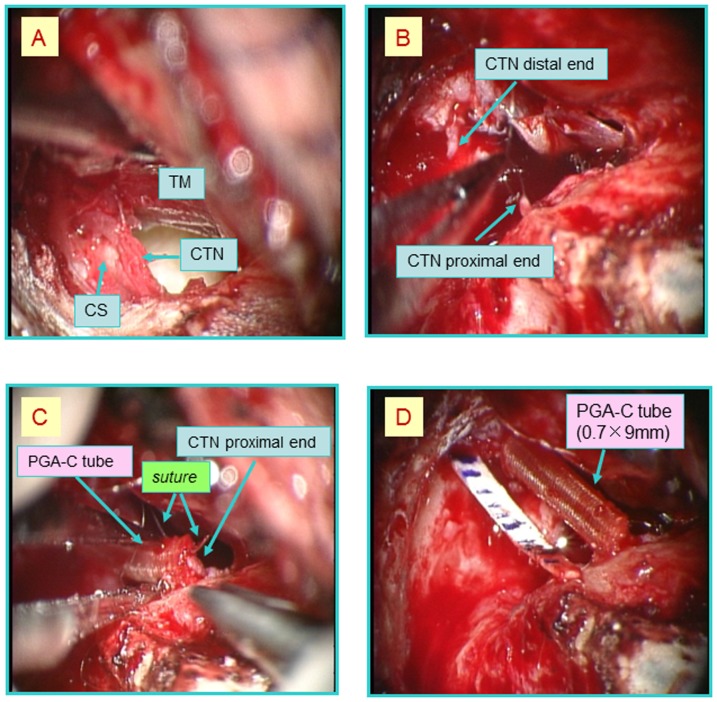
Intraoperative images of the 1^st^ surgical procedure in patient 1. Abbreviations: CS, Cholesteatoma sac; TM, Tympanic membrane; CTN, Chorda tympani nerve. (A) The cholesteatoma sac was attached to the CTN. (B) The section of the CTN was resected together with the cholesteatoma. (C) The tube was secured to the proximal and distal nerve ends with epineural 10-0 polypropylene monofilament sutures. (D) Intraoperative view after brigde of a PGA-collagen tube.

The patient underwent surgery to remove the cholesteatoma. During surgery, we found that a cholesteatoma sac occupied the attic of the ear and antral cavity, and ossicles including the malleus and incus had been destroyed. The cholesteatoma sac was attached to the CTN, and dissection of the sac suggested that it had invaded the nerve fibers, as indicated in Figure 6A. Since the CTN was attenuated and had adhered to the lateral aspect of the sac, the decision was made to section the nerve in order to remove the cholesteatoma sac. The CTN that was attached to the cholesteatoma sac was resected together with the cholesteatoma (Figure 6B). The resultant seven mm nerve gap was reconstructed with a PGA-collagen tube (nine mm in length and 0.7 mm in diameter). Both the proximal and distal stumps of the severed nerve were inserted into the PGA-collagen tube to a depth of one mm, and the tube was secured to the proximal and distal nerve ends with epineural 10-0 polypropylene monofilament sutures (Prolene, Ethicon, Somerville, NJ, USA) (Figure 6C,D).

The patient's preoperative EGM threshold scores of 12 dB on the affected side, which was indicative of objective gustatory mild disturbance due to cholesteatoma, was elevated to 16 dB at 1 day and 32 dB at 1 week after the CTN resection and reconstruction (Figure 3A). However, the threshold started to decrease within ten days and had improved to within the normal range at two postoperative weeks, while the EGM threshold on the intact side displayed little change throughout the patient's postoperative course (Figure 3A). Afterwards, his EGM threshold remained normal at 18 postoperative months although he has never come to our clinic until 18 months after the 1^st^ surgery. (Figure 3B).

**Figure 7:**
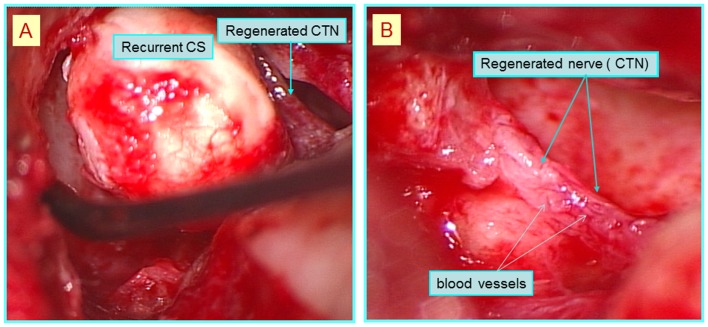
Intraoperative images of the 2^nd^ surgical procedure in patient 1. (A) The cholesteatoma sac was pressing upon and stretching the regenerated fibers. (B) The PGA-collagen tube installed during the first surgery had been absorbed and replaced by new nerve fibers with blood vessels on their surface.

Since he suffered taste disturbance at 22 postoperative months due to cholesteatoma recurrence, which was detected by CT, tympanoplasty was performed to the remove the lesion at 2 years. During surgery, we found a restiform strand running through the previous PGA-collagen tube implant site, which was assumed to be the regenerated nerve fibers. The cholesteatoma sac was pressing upon and stretching the regenerated fibers (Figure 7A). The PGA-collagen tube installed during the first surgery had been absorbed and replaced by new nerve fibers with blood vessels on their surface (Figure 7B). The sac was successfully removed, thereby releasing the pressure on the regenerated nerve fibers. The patient's EGM threshold improved from preoperative scores of 36 to 18 dB at two weeks after the second surgery (Figure 3B).

## Discussion

The artificial nerve conduit used in this study was proven to aid the regeneration of the chorda tympani nerve, which does not have any floor below it to support grafting, after it had been severed. This is the first report to describe the successful regeneration of a nerve running through an aerial space. The artificial nerve conduit, PGA-collagen tube is composed of the outer tube made of a PGA mesh and the inner lumen filled with a spongiform collagen, which provide a favorable microenvironment for nerve progression [10], [11]. The PGA tube is considered to retain its shape for a sufficiently long period to achieve nerve regeneration through the collagen sponge of the inner lumen which dissolves in the body [10], [12]. Moreover, a conduit framework has been suggested to help to direct the nerve fibers in the right orientation and guide the regenerating nerve to the intended target [17]. In the present study, the PGA-based tube framework probably played a role in preventing the nerve fibers from being misdirected during the regeneration process and guiding them correctly towards the opposite cut nerve ends through the collagen sponge-filled tube lumen, as the nerve fibers successfully extended and regenerated in the correct direction despite passing through the air and not being supported by a scaffold.

To take advantage of the features of this PGA-collagen tube, some experimental studies and clinical trials have been extended to examine the performance of the PGA-collagen tube during nerve gap reconstruction. In a previous study, a histological examination performed at 4 months after the implantation of a PGA-collagen tube detected the regeneration of nerve tissue structures, including myelinated axons and Schwann cells. In electrophysiological studies, electrophysiological analysis (e.g., of somatosensory evoked potentials and electromyograms) demonstrated functional recovery of the regenerated nerves at a growth rate of 1–2 mm/day [10], [12]. In addition, PGA-collagen tubes have recently been applied to clinical cases involving peripheral nerve defects, such as those involving the proper digital nerve or superficial peroneal nerve, and achieved successful results including symptomatic and functional recovery [13], [14]. In this study, the reconstruction of a severed CTN in the aerial space within the tympanic cavity using a PGA-collagen tube resulted in successful recovery in terms of objective taste function which was assessed using EGM.

Few studies have reported on the time course of the findings of EGM, a quantitative gustatory function test [15], [16], after the CTN has been severed. One study reported that the EGM threshold increased rapidly at two weeks after the CTN had been sectioned and did not return to the baseline level during the follow-up period. Thirty percent or fewer of the cases demonstrated partial recovery after two years [18]. Another report suggested that one to two years are needed until the patient's EGM threshold recovers and stabilizes [19]. Thus, although it is difficult to determine how long it takes for taste disturbance to recover after CTN resection, it seems to take at least 1 year. According to these reports, the mechanism underlying the recovery of taste after unilateral CTN section seems to be associated with neuronal innervation to the lesional side from the contralateral intact side and/or from the ipsilateral glossopharyngeal nerve [3], [20], [21], [22], [23]. Furthermore, since nerve growth is suggested to occur at a rate of 1–2 mm day, the recovery should require 7–14 days at least for 7 mm nerve gap [24]. In this study, gustatory function as assessed by EGM exhibited early improvements at two weeks after the reconstruction of the severed CTN with a PGA-collagen tube, and these measures detected complete recovery within three months in all patients. The times of the recovery onset observed in all patients appear to be consistent with nerve growth. Therefore, the prompt improvement of gustatory function appears to have been caused not by innervation from other healthy regions such as the contralateral CTN or ipsilateral glossopharyngeal nerve but by the regeneration of the nerve itself through the PGA-collagen tube.

In patient 1, an index case, during the second surgical procedure, a restiform strand covered with blood vessels, which seemed to be composed of nerve fibers, was detected at the PGA-collagen tube implantation site. There are reports describing that the reconstructed site with PGA collagen tube had been replaced by a white linear tissue on gross view in animal experiments. This observation, which could be viewed macroscopically, has been suggested to be an evidence for regenerated nerve fibers, and hence could support our surgical macroscopic finding for suggesting the CTN regeneration [10], [11].

As the cholesteatoma sac that was pressing down on the regenerated nerve was removed, the patient's gustatory function returned to normal in the early postsurgical course. Therefore, the morphological nerve fibers seen during the second surgical procedure probably had the potential to transmit gustatory information and represented a functionally regenerated CTN. The results from our second look surgery strongly suggest that reconstruction of CTN with a PGA-collagen tube in our first surgery allows a regeneration of the nerve.

Furthermore, no adverse events, including middle or inner ear dysfunction causing hearing loss, tinnitus or dizziness were observed in any of the patients due to the reconstruction with PGA-C tube. PGA, a bioabsorbable substance, generally used as surgical suture material have been proved to be safe although PGA materials have not been applied in the otologic surgery. It was reported that Poly-lactic/glycolic acid placed on the round window niche for drug delivery to the inner ear had no significant adverse effects in the middle and inner ear except for temporary hearing loss [25]. It is unlikely that PGA-collagen tube affects middle and inner ear and cause dysfunctions therein.

## Conclusion

These results suggest that reconstruction of the CTN with an artificial nerve conduit, a PGA-collagen tube, allows functional and morphological regeneration of the nerve and facilitates the complete recovery of taste function. Therefore, PGA-collagen tube might be a useful alternative device for a repair of the CTN section during middle ear surgery. This is the first report to describe the successful regeneration of a nerve running through an aerial space. However, further research compared with control is required to confirm these preliminary results obtained from low number of patients.
